# Assessment of neighborhood street characteristics related to physical activity in the Lower Mississippi Delta

**DOI:** 10.15171/hpp.2019.03

**Published:** 2019-01-23

**Authors:** Jessica L. Thomson, Melissa H. Goodman, Alicia S. Landry

**Affiliations:** ^1^US Department of Agriculture, Agricultural Research Service, Stoneville, MS, USA; ^2^Department of Family and Consumer Sciences, University of Central Arkansas, Conway, AR, USA

**Keywords:** Neighborhood, Built environment, Physical activity, Safety

## Abstract

**Background:** Physical activity levels were low for pregnant and postpartum participants in a diet and physical activity intervention. To explore micro level characteristics of participants’neighborhoods related to physical activity, an ancillary study was conducted.

** Methods: ** This cross-sectional study encompassed the neighborhood street segments of women participating in a diet and physical activity intervention that was conducted in the Lower Mississippi Delta. A neighborhood was defined as all street segments within one-fourth walking mile of a participant’s home address. Street segments were measured using the Rural Active Living Assessment’s Street Segment Assessment tool. In the field and on foot, raters measured street segments using neighborhood maps with segments identified.

**Results:** Mean street segment length was 0.22 miles (SD = 0.14). All segments had flat terrain with residential (98%), open spaces (74%), and public/civic (34%) as the most prevalent land uses. Almost three-fourths of segments did not have any sidewalks (69%), sidewalk buffers or defined shoulders (73%), crosswalks or pedestrian signage (69%), or posted speed limits (74%).However, 88% had stop signs and almost all (96%) had street lighting and were paved multi lane roads (95%) with low traffic volume (90%). Most residential structures present were single family detached homes (95%) and the most common public/civic and commercial structures were churches (24%) and convenience stores (9%), respectively. Almost all of the street segments were rated as walk able (99%) and aesthetically pleasing (94%).

**Conclusion:** Neighborhood street segments surrounding Delta Healthy Sprouts participants’homes were walk able and aesthetically pleasing. However, safety features such as sidewalks,pedestrian signage, and posted speed limit signs were lacking. To address these inadequate pedestrian safety features, infrastructure changes are needed for small rural towns.

## Introduction

Walking, the most common form of physical activity in the United States, is an excellent way for individuals to become more active and improve their health.^1^ Yet among women, some of the lowest prevalence for walking is reported among those with obesity, non-Hispanic African Americans, and residents of the South.^2^ Additionally, in the United States, residents of rural communities walk less than their urban counterparts.^3^ Efforts to increase the percentage of adults who walk, particularly minority women living in rural areas, could lead to more adults meeting physical activity guidelines, thereby reducing the burden of chronic diseases and premature deaths associated with low levels of physical activity.^2^

The built environment can affect physical activity levels of community residents, both positively and negatively. In rural towns, pedestrian safety features, such as the presence of crosswalks and light signals and slow traffic speeds are perceived characteristics that have been positively associated with utilitarian walking, while positively associated geographical features include the presence of manufacturing land use and transit stops and close proximity to a neighborhood school.^4^ Further, lower income rural adults who reported neighborhood streets as places for exercise were more likely to meet recommendations for physical activity than their counterparts who reported having no place to exercise.^5^ Thus, rural neighborhoods containing streets with characteristics that promote physical activity may be an important support for encouraging exercise, particularly walking, among rural residents.

Designed to test the comparative impact of two maternal, infant, and early childhood home visiting curricula on health behaviors of pregnant women and their infants, Delta Healthy Sprouts was conducted between 2013 and 2016 in the rural Lower Mississippi Delta region of the United States.^6^ Physical activity was a health behavior targeted for improvement and while walking was not the only form of exercise promoted during the intervention, it was the most commonly recommended. However, based on analysis of the trial’s physical activity data, it was concluded that maternal physical activity levels were low at baseline and did not improve in either the gestational or postnatal periods for this cohort of rural, Southern, primarily African American women.^7,8^ Thus, to explore physical activity related characteristics of these women’s neighborhood environments, an ancillary investigation, the Delta Neighborhood Physical Activity Study, was implemented. Researchers hypothesized that the neighborhood environments of Delta Healthy Sprouts participants were not optimal for physical activity participation, particularly walking for either utilitarian purposes or for exercise.

## Materials and Methods

### Study design and setting

An ancillary cross-sectional study to the Delta Healthy Sprouts comparative impact trial, the Delta Neighborhood Physical Activity Study included the 12 towns in which Delta Healthy Sprouts participants resided. Data collection began in August 2016 and was completed in September 2017.

### Measures

Using the Rural Active Living Assessment (RALA) tools, the built environments of the 12 towns were measured. These three observational tools are designed to assess physical environmental features and amenities, town characteristics, and community programs and policies that can affect physical activity among residents of rural communities.^9^ The Program and Policy Assessment tool consisted of 20 questions that provided an inventory of each town’s programs and policies related to physical activity. The Town-Wide Assessment tool consisted of 18 town characteristic questions and an inventory of 14 recreational amenities that measured each town’s physical characteristics on a broad (macro) level. Results from the Program and Policy Assessment and Town-Wide Assessment are being reported in another paper currently under in press.

The Street Segment Assessment tool, used to measure Delta Healthy Sprouts participants’ neighborhoods, consisted of 28 questions that measured street segment characteristics on a detailed (micro) level. A neighborhood was defined as all street segments falling within one-fourth walking mile by road of a participant’s home address.^10^ Survey items included primary land use and terrain, walkability characteristics (e.g., sidewalks, buffers and shoulders, crosswalks), land use structures (e.g., homes, public/civic buildings, commercial structures, schools), subjective assessment of walkability and aesthetics, and general conditions (i.e., weather, season, and day of week). Even though two of the towns exceeded the recommended population size (<10 000) for use of the RALA tools, they were measured with these surveys to maintain consistency in the type of data collected. Because no scoring algorithm is available for the Street Segment Assessment tool, summary measures were used to present the data captured with this instrument.

Training for the RALA tools consisted of watching a recorded web-based seminar that discussed the three tools. Upon request, the webinar is available from the Active Living Research team. The RALA Codebook was then reviewed and discussed by senior research members with research associates (raters). Subsequently, raters were trained to use the Street Segment Assessment tool in the field. Discrepancies in measurement were discussed and resolved and field training continued until complete agreement was reached. Raters were provided with neighborhood maps to use in the field that contained street segments and their unique identifiers. Data collection occurred on foot by pairs of raters using tablets loaded with electronic versions of the RALA tools. All three RALA tools were recreated as electronic surveys using Snap Surveys software (version 11.20, Snap Surveys Ltd., Portsmouth, NH). Data were stored on the Snap WebHost, an online, mobile, secure survey management system.

### Data analyses

Statistical analysis was performed using SAS® (version 9.4, SAS Institute Inc., Cary, NC). Descriptive statistics – frequencies, percentages, means, and standard deviations – were used to summarize street segment characteristics. Geographic analysis (i.e., mapping) was performed using ArcGIS (version 10.4, Esri, Broomfield, CO) and Google Maps. Using the distance capabilities of these systems, each participant’s home was graphically located and all single block segments within one-fourth mile distance by road were pinpointed. Subsequently, segments were built by combining blocks of the same street until the one-fourth walking mile was reached. Segments started at a cross street and ended at a cross street unless the one-fourth mile walking distance occurred prior to reaching the cross street or a natural barrier (e.g., field, stream, dead-end) or man-made barrier (e.g., ditch) was encountered. Busy roads and highways also were considered man-made barriers if their intersection was uncontrolled (i.e., did not contain a 4-way stop or traffic light) or no sidewalks were present. Each street segment was given a unique identifier. Street segment length was verified physically in the field by research associates using the Track My Walks app and subsequently confirmed using ArcGIS. Street segments contained in multiple study participants’ neighborhoods were measured only once but associated with all appropriate participants.

## Results

**Figure 1:**
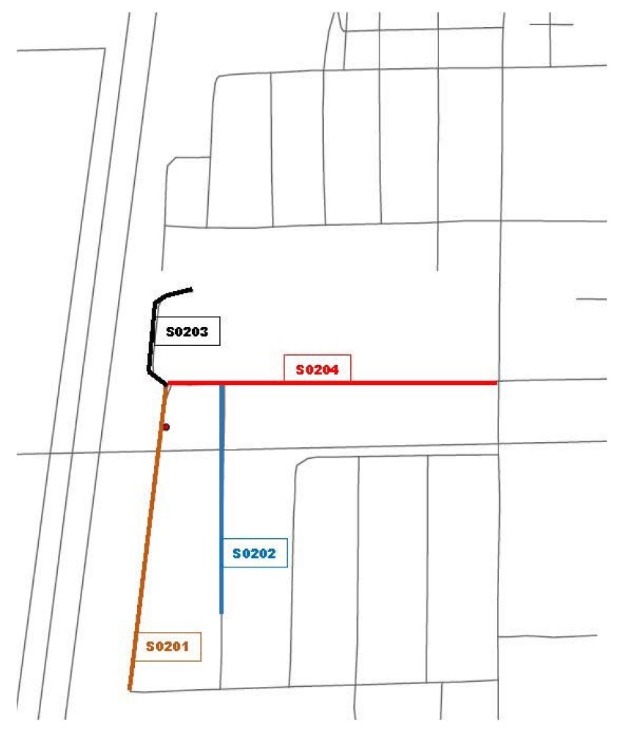
Example of a standard neighborhood included in the Delta Neighborhood Physical Activity Study. This map illustrates the street segments of a neighborhood located in primarily residential section of town. The red circle depicts a Delta Healthy Sprouts participant’s residence and the colored lines with identifying numbers depict the neighborhood street segments that were measured.

In total, 615 street segments were measured. The median length of the street segments was 0.19 miles (mean = 0.22, SD = 0.14). Presented in Figures 1 through 4 are maps that represent different types of neighborhoods that were measured. As can be seen in these figures, the types of neighborhoods in which Delta Healthy Sprouts participants resided varied with respect to the number of segments and presence (or absence) of features.

**Figure 2:**
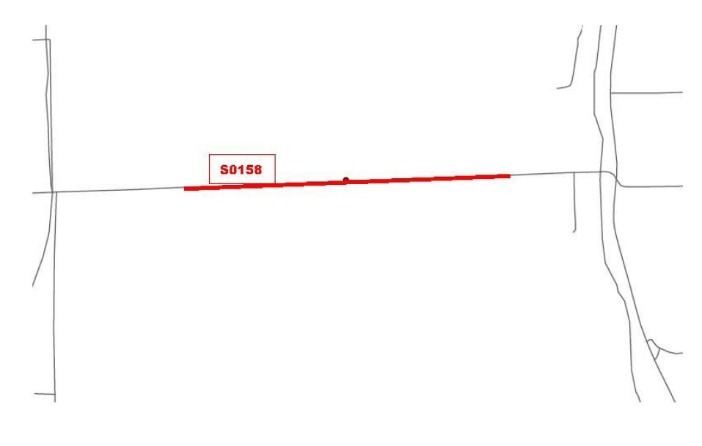
Example of a rural neighborhood included in the Delta Neighborhood Physical Activity Study. This map illustrates the street segment of a neighborhood located outside town limits. The red circle depicts a Delta Healthy Sprouts participant’s residence and the colored line with identifying number depicts the neighborhood street segment that was measured.

**Figure 3:**
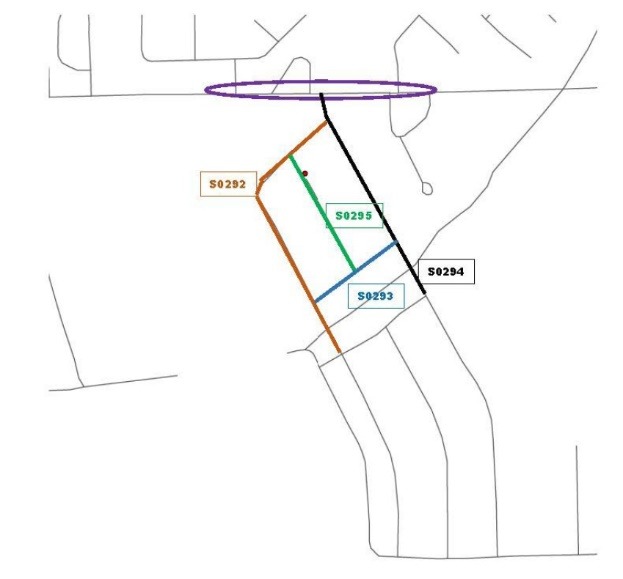
Example of a man-made barrier neighborhood included in the Delta Neighborhood Physical Activity Study. This map illustrates the street segments of a neighborhood bounded by a busy street that inhibits walking due to its uncontrolled intersection and lack of sidewalks. The red circle depicts a Delta Healthy Sprouts participant’s residence, the colored lines with identifying numbers depict the neighborhood street segments that were measured, and the purple ellipse depicts the busy street that functions as a barrier.

**Figure 4:**
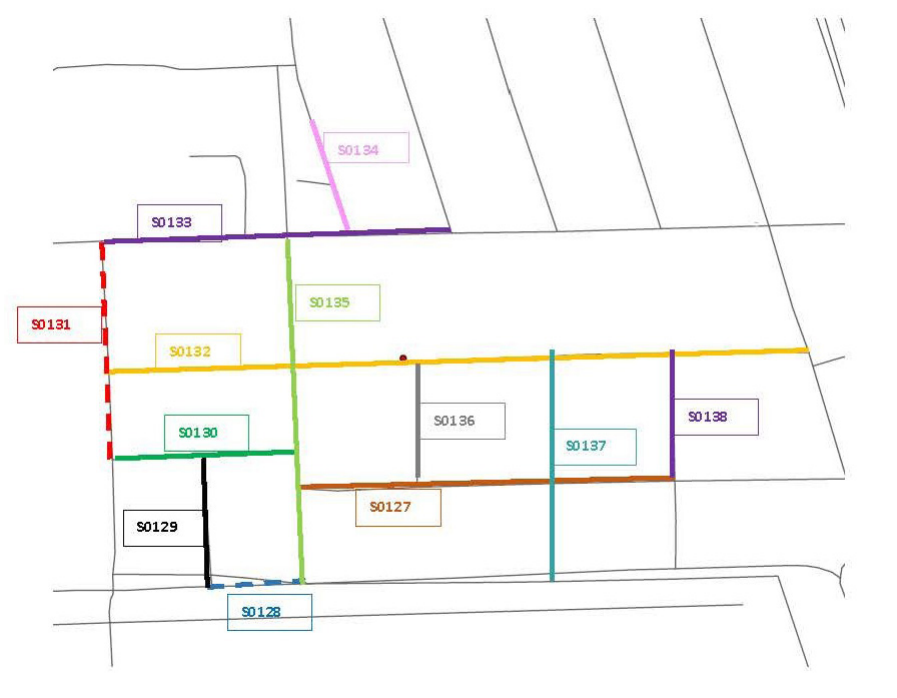
Example of a natural barrier neighborhood included in the Delta Neighborhood Physical Activity Study. This map displays the street segments of a neighborhood bounded by an agricultural field containing turn rows (i.e., areas at end of field for turning around farm equipment). The red circle depicts a Delta Healthy Sprouts participant’s residence, the solid colored lines with identifying numbers depict the neighborhood street segments that were measured, and the dotted colored lines with identifying numbers depict the turn rows that were not measured.

Presented in Table 1 are the street segment characteristics measured by the RALA Street Segment Assessment tool. All segments had flat terrain and the most prevalent land use purposes were residential (98%), followed by open spaces (74%), public/civic (34%), and commercial (27%). Almost three-fourths of the street segments did not have any sidewalks (69%), sidewalk buffers or defined shoulders (73%), signage (crosswalk, pedestrian, or children at play) (69%), or posted speed limits (74%). However, 88% had stop signs and almost all (96%) had street lighting and were paved multi-lane roads (95%) with low traffic volume (90%). Most of the residential structures present on the street segments were single family detached homes (95%) and the most common public/civic structures present were churches (24%) followed by athletic fields/courts (9%) and playgrounds (8%). The most common commercial structures present were convenience stores (9%) followed by restaurants/cafés (3%). Percentages of walkability items in good or excellent condition ranged from 85% for roads to 100% for signage, traffic lights, speed bumps, and public lighting (data not shown). Similarly, for the majority of residential, public/civic, and commercial structures, 100% of them were in good or excellent condition with the following exceptions – residential: mobile homes (89%), single family detached homes (90%), and multi-family homes/apartments (92%); public: playgrounds (96%) and churches/other religious buildings (99%); commercial: convenience stores (91%) and other commercial buildings (94%) (data not shown). All elementary and middle/junior high and 90% of high schools were in good or excellent condition (data not shown). Almost all of the street segments were rated as walkable (99%) and aesthetically pleasing (94%) by research data collectors.

## Discussion

Presented in this paper are physical activity related characteristics, including walkability aspects, of neighborhood street segments surrounding Delta Healthy Sprouts participants’ homes. Results indicate that neighborhood walkability safety features, including the absence of sidewalks, buffers or defined shoulders, pedestrian and speed limit signage, and connectivity to other sections of town, may have played a role in the low levels of physical activity observed in this cohort of Southern, primarily African American women living in rural towns. Less than one-fourth of the street segments had sidewalks on both sides of the street, although 98% of the sidewalks in the present study were in good or excellent condition. Similar results were reported in a study conducted in Deep South rural towns for which approximately one-fourth of street segments had sidewalks,^11^ while a study conducted in rural, predominantly Latino towns in Washington reported close to half of street segments had sidewalks, although only one-third were in good condition.^12^ Conversely, only 13% of street segments had sidewalks on both sides of the street in a study conducted with rural towns in Hawaii, although over one-third of the segments had crosswalks.^13^ In the present study, none of the street segments had crosswalks, very few had a traffic light, and less than one-fifth had posted speed limit signs of 25 miles per hour or less. These combined results suggest that street safety features that have been associated with any walking and/or higher levels of walking^4^ may be missing in rural towns throughout the United States.

A systematic review of correlates of physical activity found that aesthetics is consistently associated with physical activity in adults.^14^ Results from previous studies conducted with rural towns in the United States have yielded mixed results concerning aesthetics of street segments. Most (88%) of street segments were rated as aesthetically pleasing in the Deep South study,^11^ while only half (49%) were rated likewise in the Washington study conducted in Latino communities.^12^ In the present study, physical activity levels of Delta Healthy Sprouts participants were low despite over 90% of their neighborhood street segments receiving high ratings for walkability and aesthetics. These results suggest that lack of safety features may have played a larger role in the decision to be physically activity than did aesthetics of neighborhood street segments in this cohort of Southern, primarily African American women residing in rural, Lower Mississippi Delta towns. Supporting this supposition, authors of a study designed to explain physical activity through examination of interactions between perceived safety and built environment variables stated that neighborhood pedestrian safety may be one of the more important factors associated with an individual’s leisure walking and overall physical activity.^15^

According the 2017 status report for *Step it Up!*, The Surgeon General’s Call to Action to Promote Walking and Walkable Communities, the state of Mississippi has adopted a Complete Streets policy that can help create more walkable communities.^1^ However, Mississippi has less than 11 such policies at local or regional levels, no combined bicycle and pedestrian master plan (or two stand-alone plans), and less than 15% of its schools participate in Safe Routes to School programs.^1^ A multi-state evaluation of active school travel found that walking and bicycling increased in four target states (including Mississippi) after implementation of Safe Routes to Schools programs.^16^ Although 45% of Delta Healthy Sprouts participants lived within one-half mile of a public school and 70% lived within one mile, only one of the 12 towns in which participants resided participated in a Safe Routes to School program. Thus, the implementation of such programs has the potential to impact not only children’s physical activity but also that of the parents who choose to walk or bike with their children to school. Clearly action is still needed by the state to improve and promote walking and walkable communities, particularly in rural areas.

Despite the known benefits of exercise during pregnancy, over 90% of Delta Healthy Sprouts participants engaged in low amounts of physical activity during their first trimester of pregnancy and none met the recommended 150 minutes per week of moderate intensity physical activity.^17^ Results from a maternal prenatal walking program indicated that both low and vigorous intensity walking are safe and beneficial to mothers and their infants.^18^ However, a systematic review of physical activity and pregnancy reported lack of access to suitable areas for physical activity as an environmental barrier.^19^ It may be that walking among residents of rural communities, including pregnant women, will not increase until infrastructure changes occur that make walking both safe and convenient for all individuals.

Strengths of this study include the use of a validated and objective tool to measure street segment characteristics associated with physical activity as well as the population studied. Southern, African American adults residing in rural communities are less likely to achieve recommended amounts of physical activity as compared to their counterparts.^20-22^ However, the nonrandom selection of street segments may limit the generalizability of the study’s results to other rural communities. Further, the potential influence of Delta Healthy Sprouts participants’ personal health and psychosocial characteristics bears mentioning. Before becoming pregnant, two-thirds of the women were classified as overweight or obese and they scored relatively low for physical activity self-efficacy at baseline.^17^ Subsequently, mean post-pregnancy body mass index was in the obese range and physical activity self-efficacy remained low throughout the postnatal period.^8^ Both overweight/obesity and low self-efficacy for participating in physical activity have been negatively associated with participation in physical activity.^14^ Due to the relative homogeneity in the amount of physical activity performed by Delta Healthy Sprouts participants, it is difficult to separate the effects of individual participant characteristics from environmental factors at the neighborhood street level in terms of their influence on physical activity.

## Conclusion

Neighborhood street segments surrounding Delta Healthy Sprouts participants’ homes were walkable and aesthetically pleasing. However, safety features such as sidewalks, pedestrian signage, and posted speed limit signs were lacking. Safe neighborhood walking routes are needed to encourage and facilitate walking for utilitarian purposes as well as for exercise among residents of rural communities. To address inadequate pedestrian safety features often present in rural towns, infrastructure changes are needed.

## Ethical approval

The Delta Neighborhood Physical Activity Study was approved and classified as exempt by the Institutional Review Board of Delta State University (IRB protocol number 16-028).

## Competing interests

The authors declare that they have no competing interests. The views expressed are solely those of the authors and do not reflect the official policy or position of the United States government or the authors’ affiliated institutions.

## Funding

This research was funded by the United States Department of Agriculture, Agricultural Research Service (Project 6401-53000-003-00D).

## Authors’ contributions

JLT conceptualized and designed the study, conducted the statistical analyses, drafted the initial manuscript, and reviewed and revised the manuscript. MHG conceptualized and designed the study, conducted the geographical analyses, and critically reviewed the manuscript for important intellectual content. ASL conceptualized the study and critically reviewed the manuscript for important intellectual content.

## Acknowledgments

The authors thank Debra Johnson and Donna Ransome for their research support.

**Table 1:** Characteristics of neighborhood street segments (N=615) in the Delta Neighborhood Physical Activity Study, 2016-2017

**Characteristic**	**No.**	**%**
Land use^a^		
Residential	603	98.0
Commercial	167	27.2
Industrial	3	0.5
Public/civic	209	34.0
Open space	453	73.7
School/school zone	2	0.3
Terrain - flat	615	100.0
Sidewalk		
Both sides of street	136	22.1
One side of street	55	8.9
Intermittent	2	0.3
None	422	68.6
Buffer and shoulder		
Sidewalk buffer	164	26.7
Defined shoulder	0	0.0
None	451	73.3
Signage		
Crosswalk or crossing signal	0	0.0
Pedestrian sign	50	8.1
Children at play sign	151	24.6
None	424	68.9
Other safety features		
Stop sign	543	88.3
Traffic light	27	4.4
Speed bump	7	1.1
Public lighting	592	96.3
None	5	0.8
Connectivity^c^ - none	615	100.0
Road type		
Paved multi-lane	581	94.5
Paved single lane	18	2.9
Unpaved	16	2.6
Posted speed limit (mph)		
10	3	0.5
15	40	6.5
20	35	5.7
25	27	4.4
30-50	57	9.3
None	453	73.7
Traffic volume		
Low	554	90.1
Medium	50	8.1
High	11	1.8
Barriers		
Highway/busy street	43	7.0
Private property	1	0.2
Natural features^c^	18	2.9
Other^d^	6	1.0
None	549	89.3
Settled density		
Dense	2	0.3
Moderate	603	98.0
Low/dispersed	10	1.6
Residential structures		
SF detached homes	583	94.8
MF homes/apartments	112	18.2
Mobile homes	72	11.7
Public/civic structures		
Library	8	1.3
Museum	3	0.5
Community center	16	2.6
Post office	7	1.1
Town office	9	1.5
Courthouse	2	0.3
Police station	6	1.0
Fire station	9	1.5
Church/other religious	146	23.7
Hospital/other health	4	0.7
Athletic fields/courts	54	8.8
Playground	48	7.8
Other^e^	8	1.3
None	401	65.2
Commercial structures		
Restaurant/café	20	3.3
Bar	9	1.5
Gas station	1	0.2
Convenience store	58	9.4
Small retail	14	2.3
Fitness center	1	0.2
Private medical office	6	1.0
Private other office	16	2.6
Other^f^	125	20.3
None	444	72.2
Schools (public)		
Elementary	19	3.1
Middle/junior high	5	0.8
High	10	1.6
Other^g^	11	1.8
None	572	93.0
Industrial/agricultural		
Light industrial	1	0.2
Farmland	20	3.3
None	594	96.6
Walkable^h^		
Strongly agree	232	37.7
Agree	376	61.1
Disagree	7	1.1
Strongly disagree	0	0.0
Aesthetically pleasing^h^		
Strongly agree	145	23.6
Agree	431	70.1
Disagree	39	6.3
Strongly disagree	0	0.0
Current weather		
Sunny/clear	508	82.6
Partly cloudy	91	14.8
Overcast	16	2.6
Season		
Winter	100	16.3
Spring	284	46.2
Summer	231	37.6
Fall	0	0.0
Weekday	615	100.0

Abbreviations: SF, single family; MF, multi-family. ^a^ More than one selection possible. ^b^ Sidewalk, bike path, trail linking to other sections of town. ^c^ Field, creek, river, or lake. ^d^ Man-made ditch, locked gate. ^e^ Boys and girls club, park, swimming pool, and recreation center. ^f^ Examples: auto body repair shop, bank, beauty/barber shop, daycare, funeral home, laundry mat. ^g^ Head Start Center, alternative school, and child development center. ^h^ Subjective assessment by raters.
